# Supplementary material for: PAX7 target genes are globally repressed in facioscapulohumeral muscular dystrophy skeletal muscle
Source: Nat Commun. 2017 Dec 18;8:2152. doi: 10.1038/s41467-017-01200-4 (PMC5735185; doi:10.1038/s41467-017-01200-4)
Supplement: Supplementary file 1 — Supplementary Information [file 41467_2017_1200_MOESM1_ESM.pdf]

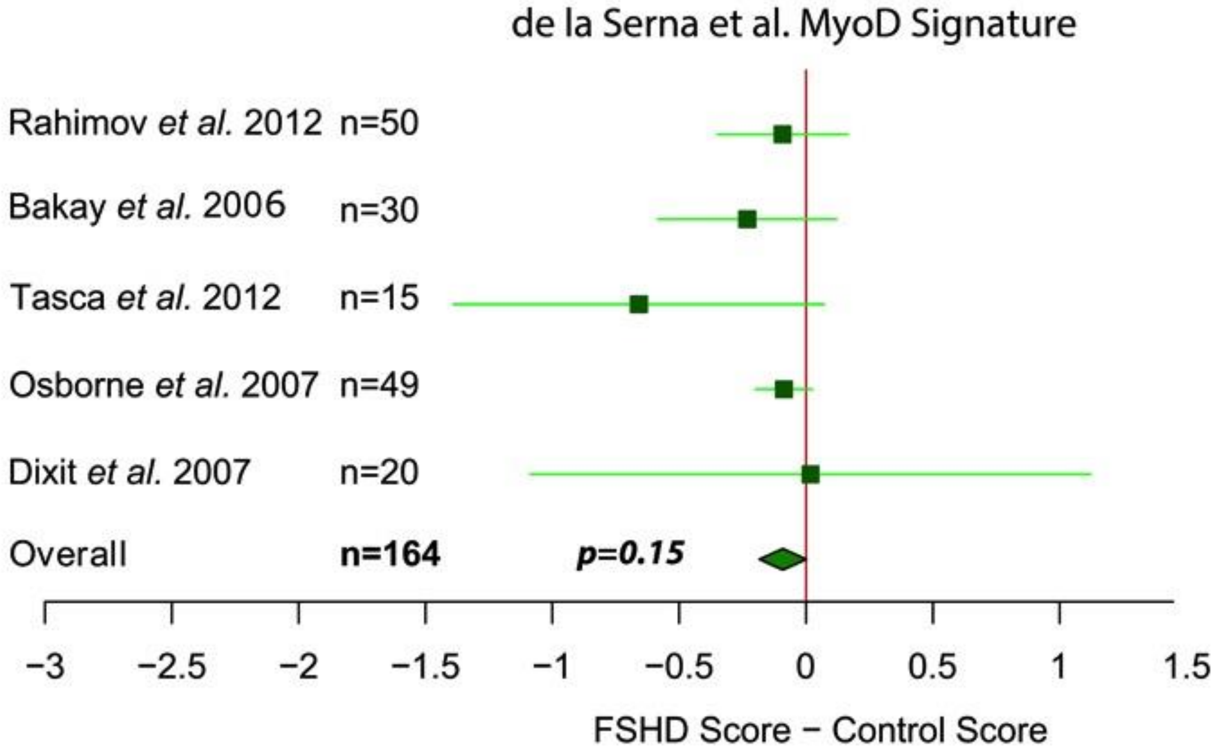

Supplementary Figure 1
MyoD target genes are not significantly altered in FSHD muscle biopsy gene expression datasets
A forest plot displays the results of meta-analysis of the discriminatory power of a MyoD target gene signature derived from de la Serna et al. 2005[1] across five published microarray FSHD muscle biopsy data sets (in total n=82 FSHD and n=82 control muscle biopsies). We observe a trend towards suppression of MyoD targets but this is not significant in any single data set nor on meta-analysis. For single studies a two tailed Wilcoxon U-test was performed to assess significance, whilst a Fisher's combined test was employed for overall assessment: either the *p*-value is given, or an asterisk would denote $p<0.05$.

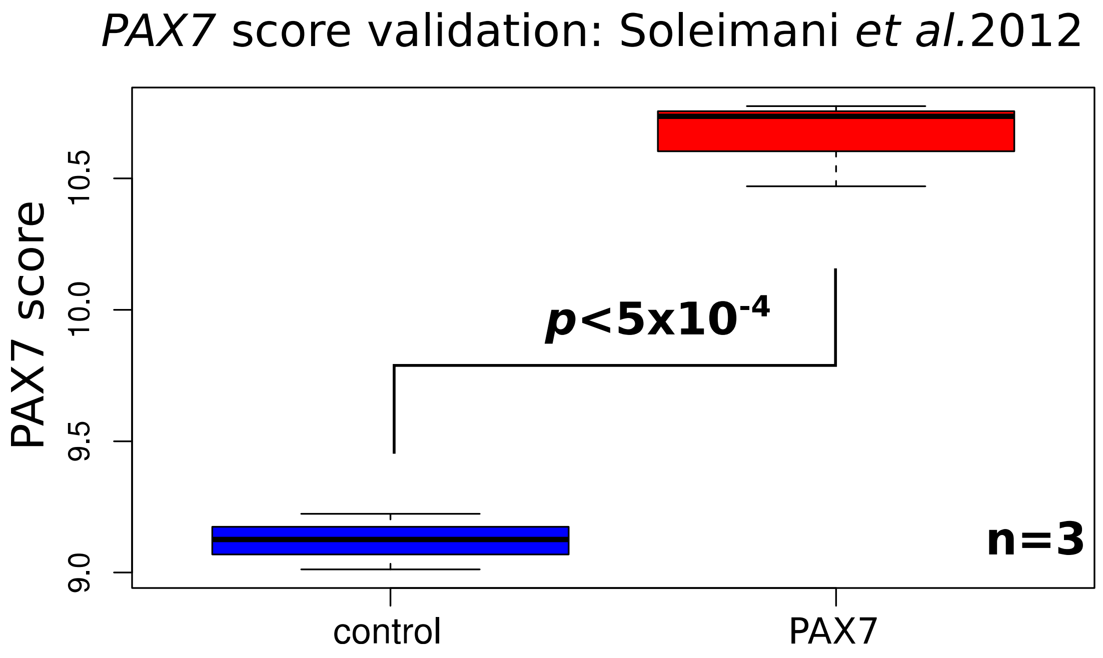

Supplementary Figure 2:
Our PAX7 target gene biomarker validates on an independent *Pax7* gene expression dataset
Our PAX7 biomarker was evaluated on a dataset published by Soleimani et al. 2012[2], profiling murine myoblasts over-expressing *Pax7* and suitable controls in triplicate. As anticipated the *Pax7* expressing samples show significantly higher levels of the PAX7 biomarker compared to controls. Boxes represents the interquartile range (IQR), with the median indicated by a line. Whiskers denote min(1.5*IQR, max(observed value)). The two-tailed unpaired t-test *p*-value is given.

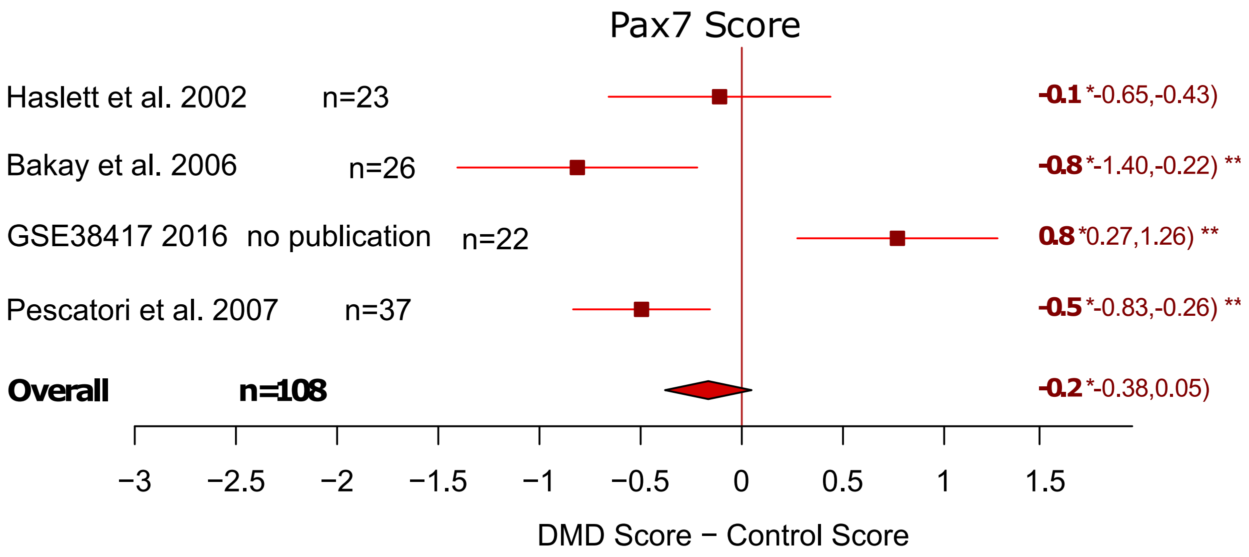

Supplementary Figure 3:
PAX7 target gene suppression in FSHD is not attributable to generalised skeletal muscle dystrophy
A forest plot displays the results of meta-analysis of the discriminatory power of the PAX7 target gene signature across four published microarray muscle biopsy data sets profiling Duchenne muscular dystrophy (DMD) against matched controls (n=108)[3-5]. The differential scores (DMD score – control score) alongside 95% confidence intervals are provided. Our PAX7 target gene signature is not a significant biomarker of DMD, demonstrating that its repression in FSHD is disease specific and not a consequence of generalised muscle pathology. For single studies a two tailed Wilcoxon U-test was performed to assess significance, whilst a Fisher's combined test was employed for overall assessment: two asterisks denote $p<0.01$.

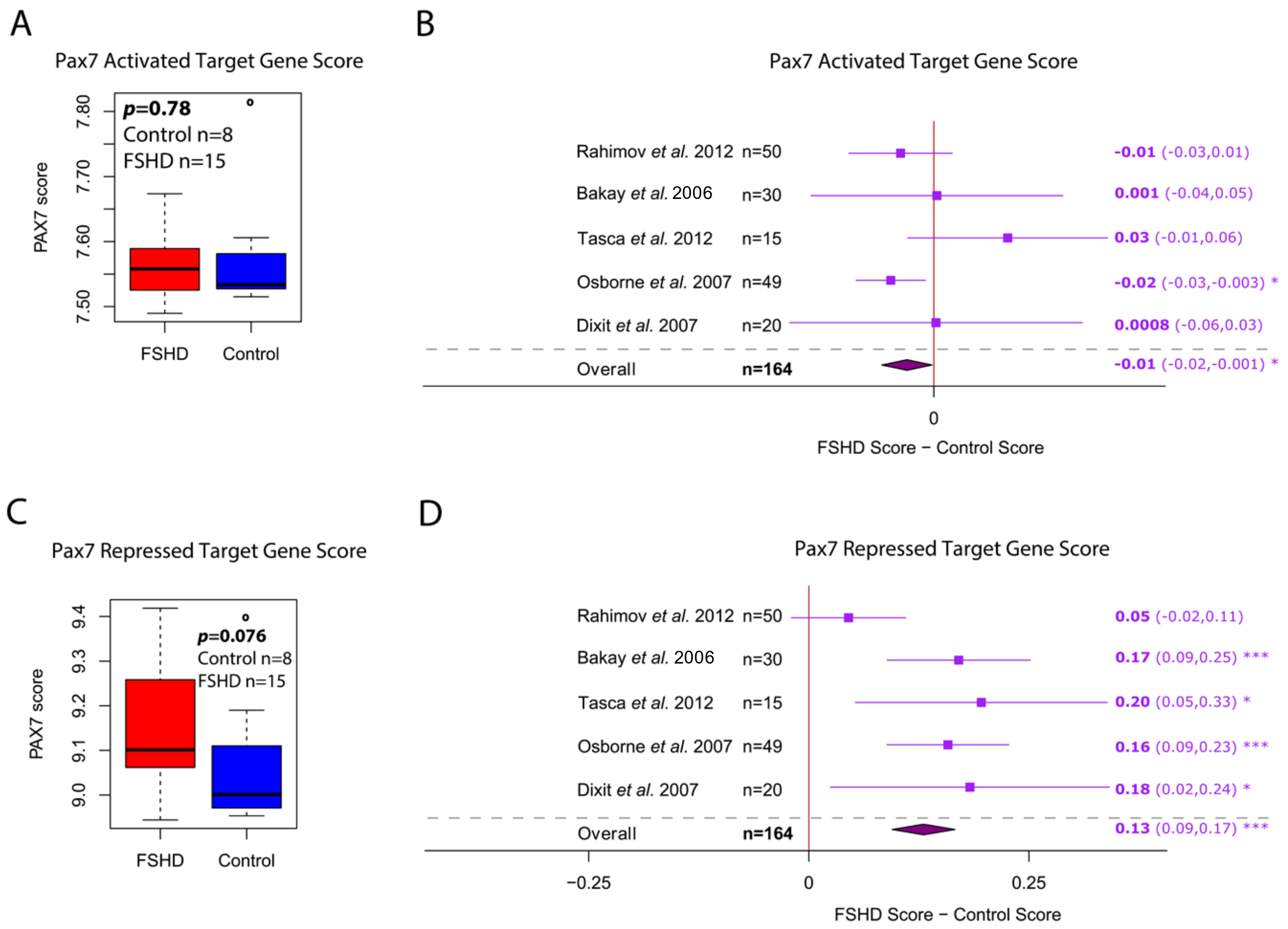

Supplementary Figure 4:
PAX7 activated or repressed target genes both contribute to the power of the PAX7 FSHD biomarker
(A) Evaluation of the PAX7 activated target genes as a biomarker of FSHD. A box plot demonstrates that the PAX7 activated target gene signature does not validate as a biomarker on the RNA-seq FSHD muscle biopsy data set published by Yao et al. 2014[6]. The box represents the interquartile range (IQR), with the median indicated by a line. Whiskers denote min(1.5*IQR, max(observed value)). 'o' represents data points greater than 1.5 IQR from the median. n=15 FSHD and n=8 control muscle biopsies.
(B) A forest plot displays the results of meta-analysis of the discriminatory power of the PAX7 activated target gene signature across five published microarray FSHD muscle biopsy data sets (in total n=82 FSHD and n=82 control muscle biopsies)[4, 7-10]. The differential scores (FSHD score – control score) alongside 95% confidence intervals are provided. The PAX7 activated target gene signature is a significant biomarker on meta-analysis.
(C) Evaluation of the Pax7 repressed target genes as a biomarker of FSHD. A box plot demonstrates that the PAX7 repressed target gene signature does not validate as a biomarker on the RNA-seq FSHD muscle biopsy data set published by Yao et al. 2014[6]. The box represents the interquartile range (IQR), with the median indicated by a line. Whiskers denote min(1.5*IQR, max(observed value)). 'o' represents data points greater than 1.5 IQR from the median. n=15 FSHD and n=8 control muscle biopsies.
(D) A forest plot displays the results of meta-analysis of the discriminatory power of the PAX7 repressed target gene signature across five published microarray FSHD muscle biopsy data sets (in total n=82 FSHD and n=82 control muscle biopsies)[4, 7-10]. The differential scores (FSHD score – control score) alongside 95% confidence intervals are provided. The PAX7 repressed target gene signature is a significant biomarker on meta-analysis.
For single studies a two tailed Wilcoxon U-test was performed to assess significance, whilst a Fisher's combined test was employed for overall assessment. *p*-values are given or an asterisk denotes $p<0.05$, ** denotes $p<0.01$ and *** denotes $p<0.001$.

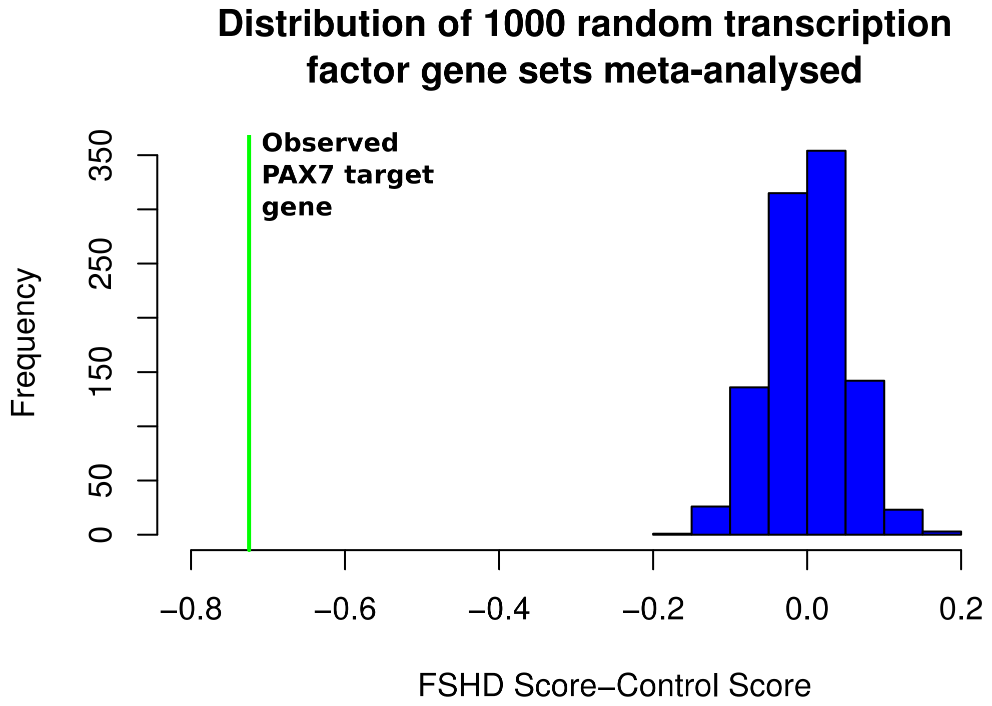

Supplementary Figure 5:
Discriminatory power of the PAX7 target gene signature on FSHD datasets is not attributable to chance
1000 gene sets of equivalent size to the PAX7 biomarker gene were selected at random and constructed into a biomarker in the same way as our PAX7 biomarker. Each randomised biomarker was then evaluated for its discriminatory power via meta-analysis across the 5 FSHD microarray muscle biopsy datasets (n=82 FSHD and n= 82 control muscle biopsies)[4, 7-10]. The distribution of combined score difference between FSHD and control samples for these 1000 random biomarkers is displayed via histogram. The value achieved by our PAX7 biomarker is shown by a green line. None of the randomised biomarkers show discriminatory power as significant as our PAX7 biomarker, hence the probability of obtaining as significant a biomarker by chance is <0.001.

References

1. de la Serna, I.L. et al. MyoD targets chromatin remodeling complexes to the myogenin locus prior to forming a stable DNA-bound complex. *Mol Cell Biol* **25**, 3997-4009 (2005).
2. Soleimani, V.D. et al. Transcriptional dominance of Pax7 in adult myogenesis is due to high-affinity recognition of homeodomain motifs. *Dev Cell* **22**, 1208-1220 (2012).
3. Haslett, J.N. et al. Gene expression comparison of biopsies from Duchenne muscular dystrophy (DMD) and normal skeletal muscle. *Proc Natl Acad Sci U S A* **99**, 15000-15005 (2002).
4. Bakay, M. et al. Nuclear envelope dystrophies show a transcriptional fingerprint suggesting disruption of Rb-MyoD pathways in muscle regeneration. *Brain* **129**, 996-1013 (2006).
5. Pescatori, M. et al. Gene expression profiling in the early phases of DMD: a constant molecular signature characterizes DMD muscle from early postnatal life throughout disease progression. *FASEB J* **21**, 1210-1226 (2007).
6. Yao, Z. et al. DUX4-induced gene expression is the major molecular signature in FSHD skeletal muscle. *Hum Mol Genet* **23**, 5342-5352 (2014).
7. Tasca, G. et al. Different molecular signatures in magnetic resonance imaging-staged facioscapulohumeral muscular dystrophy muscles. *PLoS One* **7**, e38779 (2012).
8. Dixit, M. et al. DUX4, a candidate gene of facioscapulohumeral muscular dystrophy, encodes a transcriptional activator of PITX1. *Proc Natl Acad Sci U S A* **104**, 18157-18162 (2007).
9. Osborne, R.J., Welle, S., Venance, S.L., Thornton, C.A. & Tawil, R. Expression profile of FSHD supports a link between retinal vasculopathy and muscular dystrophy. *Neurology* **68**, 569-577 (2007).
10. Rahimov, F. et al. Transcriptional profiling in facioscapulohumeral muscular dystrophy to identify candidate biomarkers. *Proc Natl Acad Sci U S A* **109**, 16234-16239 (2012).
