# Supplementary material for: PAX7 target genes are globally repressed in facioscapulohumeral muscular dystrophy skeletal muscle
Source: Nat Commun. 2017 Dec 18;8:2152. doi: 10.1038/s41467-017-01200-4 (PMC5735185; doi:10.1038/s41467-017-01200-4)
Supplement: Supplementary file 3 — Description of Additional Supplementary Files [file 41467_2017_1200_MOESM3_ESM.pdf]

Description of Supplementary Files

File name: Supplementary Data 1
Description: Genes up-regulated by DUX4 in induced human iDUX4 myoblasts described by Choi et al. 2016. Genes which are up-regulated in DUX4 expressing samples (FDR<0.05, log FC>2) in the RNA-seq data described in Choi et al. 2016[34], profiling induced iDUX4 human myoblasts after 8 hours compared to uninduced controls.

File name: Supplementary Data 2
Description: Genes up-regulated by DUX4 in DUX4 lentivirus infected human myoblasts described by Geng et al. 2012. Genes which are up-regulated in DUX4 expressing samples (FDR<0.05, log FC>2) in the microarray data described in Geng et al. 2012[35], profiling DUX4 lentivirus infected human myoblasts after 24 hours compared to control vector transduced controls.

File name: Supplementary Data 3
Description: Genes comprising our PAX7 target gene signature. Genes which are induced and repressed by *Pax7* over-expression (and conversely repressed and induced by Pax7-ERD over-expression) in our microarray of *Pax7* retrovirus construct transduced murine satellite cells compared to control vector transduced controls.

File name: Supplementary Data 4
Description: GSEA results for PAX7 up-regulated target genes. The top 100 gene sets associated with PAX7 up-regulated targets are presented, alongside enrichment statistics. Gene sets with inverse enrichment in PAX7 down-regulated targets are highlighted in yellow.

File name: Supplementary Data 5
Description: GSEA results for PAX7 down-regulated target genes. The top 100 gene sets associated with PAX7 down-regulated targets are presented, alongside enrichment statistics. Gene sets with inverse enrichment in Pax7 up-regulated targets are highlighted in yellow.

File name: Supplementary Data 6
Description: Description of the microarray and RNA-Seq derived gene expression datasets analysed. GEO accessions (where available) alongside study descriptions, including sample count, organism, experimental protocol and study design for the published data sets considered.
